# The impact of mass gatherings on the local transmission of COVID-19 and the implications for social distancing policies: Evidence from Hong Kong

**DOI:** 10.1371/journal.pone.0279539

**Published:** 2023-02-01

**Authors:** Pengyu Zhu, Xinying Tan, Mingshu Wang, Fei Guo, Shuai Shi, Zhizhao Li

**Affiliations:** 1 Urban Governance and Design Thrust, The Hong Kong University of Science and Technology (Guangzhou), Guangzhou, Hong Kong; 2 Hong Kong University of Science and Technology, Kowloon, Hong Kong; 3 University of Glasgow, Glasgow, United Kingdom; 4 International Institute for Applied Systems Analysis; 5 University of Hong Kong, Pokfulam, Hong Kong; Jahangirnagar University, BANGLADESH

## Abstract

Mass gatherings provide conditions for the transmission of infectious diseases and pose complex challenges to public health. Faced with the COVID-19 pandemic, governments and health experts called for suspension of gatherings in order to reduce social contact via which virus is transmitted. However, few studies have investigated the contribution of mass gatherings to COVID-19 transmission in local communities. In Hong Kong, the coincidence of the relaxation of group gathering restrictions with demonstrations against the National Security Law in mid-2020 raised concerns about the safety of mass gatherings under the pandemic. Therefore, this study examines the impacts of mass gatherings on the local transmission of COVID-19 and evaluates the importance of social distancing policies. With an aggregated dataset of epidemiological, city-level meteorological and socioeconomic data, a Synthetic Control Method (SCM) is used for constructing a ‘synthetic Hong Kong’ from over 200 Chinese cities. This counterfactual control unit is used to simulate COVID-19 infection patterns (i.e., the number of total cases and daily new cases) in the absence of mass gatherings. Comparing the hypothetical trends and the actual ones, our results indicate that the infection rate observed in Hong Kong is substantially higher than that in the counterfactual control unit (2.63% vs. 0.07%). As estimated, mass gatherings increased the number of new infections by 62 cases (or 87.58% of total new cases) over the 10–day period and by 737 cases (or 97.23%) over the 30-day period. These findings suggest the necessity of tightening social distancing policies, especially the prohibition on group gathering regulation (POGGR), to prevent and control COVID-19 outbreaks.

## Introduction

Managing the complex and unique health security risks posed by mass gatherings is challenging [1–3]. Many studies point out that mass gatherings can be associated with increased risks and amplified transmission of infectious diseases which are transmitted through respiratory routes, such as influenza, measles, and meningitis [4–8]. Under the circumstance of the COVID-19 pandemic, mass gatherings, according to the World Health Organization (WHO), amplify the spread of viruses and inhibit a country’s ability to respond [9]. It is well established that COVID-19 is transmitted between people through direct/indirect contacts, contaminated subjects and environmental factors, and the infection risks are proportional to the closeness of interactions with people who are infected [10–12]. A stricter risk assessment recommended by WHO states that even medium-sized gatherings may create conditions that enable the transmission of COVID-19 [9]. Given the high contagiousness of COVID-19, crowded gatherings can create environments conducive to virus transmission among participants and lead to subsequent dissemination within their families. When a community is experiencing an outbreak, mass gatherings may lead to a reduction in social distancing behavior and create challenges for the prevention and control of infectious diseases. Notwithstanding subsiding pandemic situations, public health officials still warn that mass gatherings, with extensive social mixing and uncertainties, has the potential to trigger a resurgence of COVID-19 infections. The risks of infections through mass gatherings are also partly determined by the type, venue, and location of mass gatherings and the demographics of participants [13–15]. The gatherings that involve closer and more extended contact and are held indoors or at overcrowded sites, such as professional conferences and musical events, may be more susceptible to infections. Other types of gatherings such as demonstrations and sporting events, which often take place in outdoor environments with better ventilation, may have lower transmission risks.

In 2020, social distancing and the exhortation to avoid social contact were adopted worldwide to contain the transmission of COVID-19. Many jurisdictions further imposed restrictions or bans on group gatherings as part of their anti-pandemic policy. In Hong Kong, the government imposed the Prohibition On Group Gathering Regulation (POGGR) from March 2020 onwards, stipulating the number of people allowed in a group and banning large-scale mass gatherings. Associated regulations are made by the Chief Executive in Council under section 8 of the Prevention and Control of Disease Ordinance (Cap. 599). Case clusters of COVID-19 were reported worldwide in parties and personal or social activities where a relatively small number of people were involved. To date, the scientific community has gained a significant understanding of how these small group gatherings in indoor settings could contribute to the virus transmission [16–18] (see for example, Furuse et al., 2020; Vuorinen et al., 2020; Moritz et al., 2021). But to what extent large-scale gatherings may contribute to the spread of COVID-19 in local communities? Although it is widely believed that mass gatherings could disrupt social distancing and exacerbate the scope of the virus transmission due to the complex patterns of social mixing at these events [2, 3, 15], empirical evidence to date is rather limited. Mat and others analyzed a single mass gathering, the four-day Sri Petaling Mosque religious event in Malaysia, and concluded that it was a key catalyst for the second wave of COVID-19 outbreak in the country [19]. A retrospective cohort study also found that the COVID-19 transmission and subsequent outbreak in Borriana, a municipality in Spain, were associated with several mass gathering events related to a traditional festival [20]. In contrast, opposite evidence and conclusions also exist. A recent study suggests that the *Black Lives Matter Public Gatherings* in the U.S. increased non-participants’ stay-at-home behavior such that it offset the increased transmission risks among protestors [21]. In summary, there is still a dearth of empirical research for performing a comprehensive assessment of the impact of mass gatherings on the transmission of COVID-19 in local communities. The purpose of this study is to provide additional empirical evidence and reduce this gap in the literature.

In this study, we pay special attention to public gatherings that occurred in Hong Kong in mid-2020 when public tension escalated due to the enactment of the National Security Law (NSL). During the COVID-19 outbreak, the coincidence of the relaxation of POGGR and public demonstrations against the NSL in Hong Kong provides us with an opportunity to study the impact of mass gatherings on the local transmission of COVID-19. Using Hong Kong as a case study, this study examines the epidemiological data to probe any correlation between mass gatherings and increases in COVID-19 local cases.

### Policy environment and public gatherings in Hong Kong

Hong Kong has a unique political status and subtle relationship with mainland China under the "One Country, Two Systems" principle, which safeguards national sovereignty while allowing Hong Kong a great degree of autonomy, retaining its own economic and administrative systems. In Hong Kong, public demonstrations are held yearly on July 1, usually organized by the pro-democracy camps [22, 23]. In 2019, Hong Kong experienced the most severe bout of social unrest in the city’s history. The *Anti-Extradition Law Amendment Bill Movement* proceeded in the form of public gatherings and demonstrations and was eventually wiped out in the same year because of a government crackdown. However, political tension again mounted between June 2020 and July 2020, after Beijing’s decision to promulgate the NSL.

Faced with the pandemic, the Hong Kong government has implemented various social distancing policies to control community transmission. The most significant is the Prohibition On Group Gathering Regulation (POGGR), first implemented on March 29, 2020, which forbids any form of gathering of more than four people in any public place. Public place is defined in the Regulation as “a place to which the public or a section of the public may or are permitted to have access from time to time, whether by payment or otherwise”. With Hong Kong experiencing social unrest over the past two years, social distancing policies not only reduced regular social gatherings such as dining but, more importantly, prohibited and restricted public gatherings and demonstrations. When the outbreak stabilized from mid-April through late June of 2020, the Hong Kong government actively reviewed and revised its social distancing policies. Under the "Suppress and Lift" strategy, the government reviewed the feasibility of relevant measures and made amendments to restrictions on mass gatherings on a timely basis. Infection risks of certain activities and premises/places, and overseas practices were also taken into account in such decision making. As the confirmed case numbers were on a decline at the time, the government decided to relax restrictions. The maximum allowable size of group gatherings under the POGGR was raised to 8 people on May 8, and then further lifted to 50 people on June 19. Modifications in these social distancing policies are reflected in the frequency and scale of mass gatherings held during the period. In fact, in relation to the relaxation of social distancing orders, different categories of population movement including transport, parks, and shopping all showed an upward trend in July 2020, according to the Google Mobility data. For inside-city population movement data provided by the Google, see https://www.google.com/covid19/mobility/.

**Fig 1:**
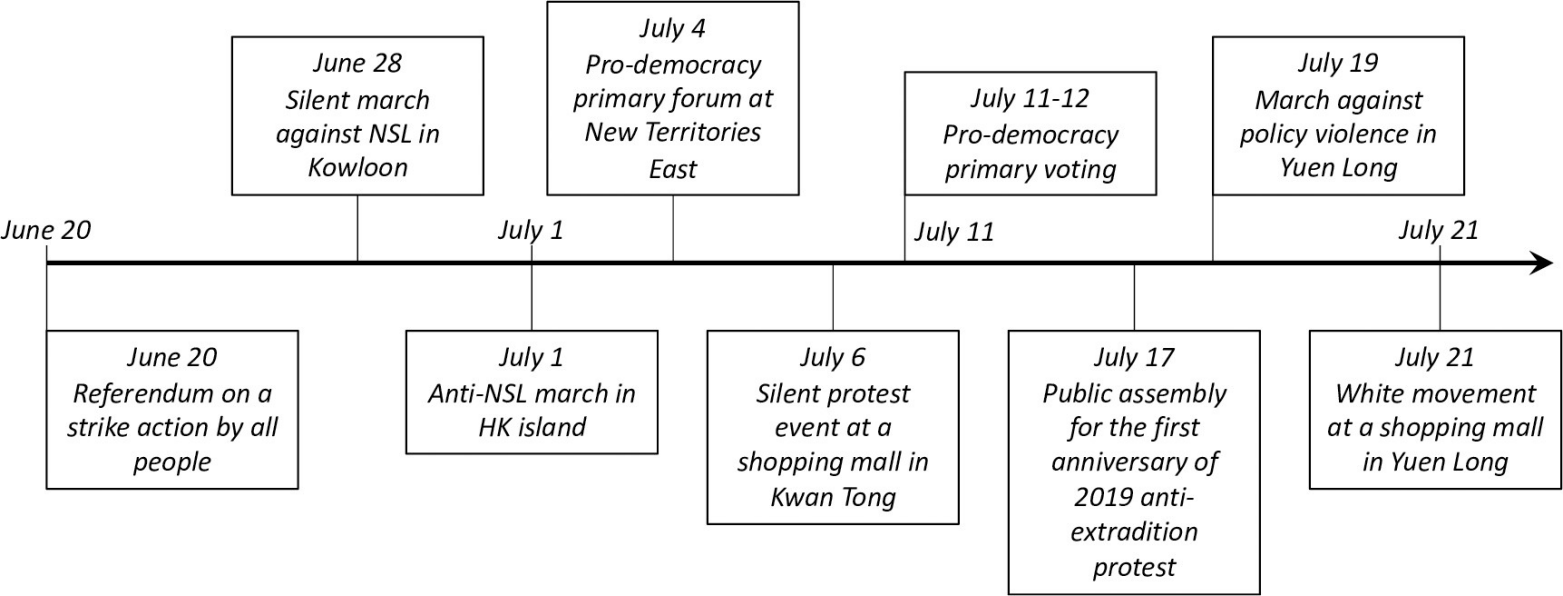
The timeline of a series of mass gatherings and public demonstrations in Hong Kong in mid-2020.

Concurrent with the relaxation of social-distancing restrictions (e.g., the maximum size for group gatherings raised to 50 people) and the enactment of the NSL, massive demonstration events reappeared in the city in late June and early July of 2020. Beginning on June 20, public gatherings and demonstrations against the NSL were frequently held in different areas of the city. Fig 1 summarizes the major gatherings that occurred during this period. The largest anti-NSL march took place on July 1, the date when anti-authoritarian public gatherings are held every year in the past. According to local media, an estimate about tens of thousands of protesters came onto the streets of Hong Kong Island to protest against the enactment of the NSL. The government reported that around 370 people were arrested by police officers [24]. Although participants in these demonstrations and protest activities usually adhered to certain precautions such as wearing masks, strictly maintaining social distancing norms was difficult since these gatherings were subject to unpredictable turns of events, including confrontation and violence. While the pandemic was still ongoing and the government’s *Stay-at-home Initiative* was still active, Hong Kong’s opposition camp organized an unofficial poll, the proclaimed pro-democratic primary election, on July 11 and 12, convening more than 20,000 people to join the offline voting. The number of valid ballots recorded throughout the two-day vote was provide by the opposition camp [25], which was not recognized by any official governmental departments. In a nutshell, the conjuncture of the lift of POGGR and the announcement of the NSL collectively triggered the emergence of public gatherings and demonstrations in Hong Kong, which could have created a risk of virus transmission. Therefore, in this paper, we try to uncover the impact of relaxing social distancing policies and the subsequent mass gathering events on COVID-19 transmission in Hong Kong. It should be noted that other forms of gatherings and social interactions had also resumed to a certain degree following the relaxation of social distancing orders back then. However, mass gatherings are characterized by greater uncertainty and risks of viral spread because of high degree of social mixing. In fact, many cases in the third wave are recorded as infection from unknow sources (comprise as high as almost 40% of total infections) [26]. There are certainly invisible transmission chains and environmental factors that contributed to the large scale of transmission in the third wave in Hong Kong. According to the Health Bureau, the third wave of outbreak mainly stems from community infections and covers a wide range of regions and industries [27]. Therefore, the research objective of this study is to investigate the overall impact of mass gatherings on the third wave of COVID-19 local outbreaks in Hong Kong after the lift of social distancing policy (i.e., the POGGR).

**Fig 2:**
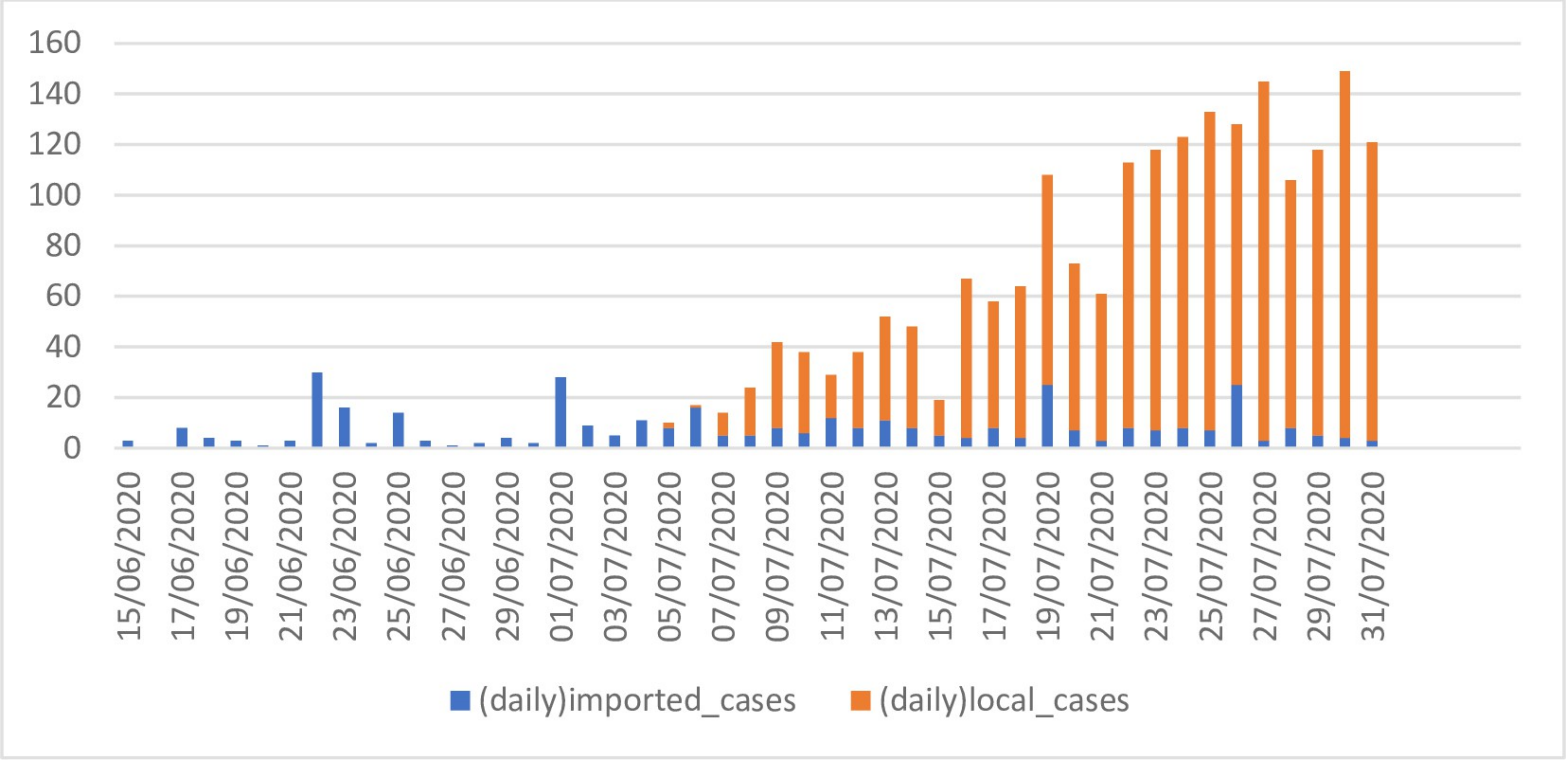
Daily COVID-19 new cases diagnosed in Hong Kong. *Source*: Centre for Health Protection under the Health Department, https://www.chp.gov.hk/en/features/102997.html.

It is also noted that Hong Kong has implemented restrictions on international and cross-border travel and imposed compulsory quarantine requirements on people coming into the city to manage the risk of case importation. Given that imported cases were largely blocked, many surmise that the rebound and catastrophic proliferation in local cases (see Fig 2) may be related to these mass gathering events and public demonstrations.

### Epidemiological trajectory of COVID-19

From mid-April to late June of 2020, the outbreak in Hong Kong had stabilized, but beginning July 5, the outbreak began to re-erupt, with an upsurge in COVID-19 infections (mostly classified as local cases). The situation suddenly deteriorated into the largest outbreak Hong Kong had then seen, which was commonly referred to as *"the third wave"* of local outbreak. The figure of daily new infections reached another peak on July 27 (145 new cases) and the city’s daily new case numbers remained in the triple digits for 7 consecutive days, bringing the city’s total number of infections to 2884 and total related deaths to 23 as of July 28. Moreover, most of the infections reported in July were local cases, and the source of infection for more than 40% of these cases was unknown [28]. Fig 2 shows the changes in new confirmed cases of COVID-19 in a daily basis in Hong Kong from June 15 to July 31, 2020. It is unclear why the pandemic in Hong Kong suddenly rebounded after the city’s initial success in controlling the outbreak. While mainland China has been able to keep the spread of COVID-19 under control since May 2020, Hong Kong and places such as the United States and Europe have seen a second or third wave of the outbreak. Yet, we are still unclear about the source and transmission routes of the third wave of the local outbreak in Hong Kong.

Given all these, this study investigates the question of whether mass gatherings, particularly in the form of public gatherings and demonstrations, contributed to the third wave of local outbreak of COVID-19 in Hong Kong. If our results suggest that mass gatherings have contributed to the increase in COVID-19 cases, it would be reasonable to strengthen social distancing measures, for example, group gathering bans, as a key strategy in containing the COVID-19 outbreak. In this regard, our study can generate useful insights on pandemic control for countries considering or starting to relax their restrictions on mass gatherings to adapt to the new normal amid this pandemic. Furthermore, our findings may help governments improve their policymaking capacity to combat other novel infectious diseases in the future. Large-scale public demonstrations in Hong Kong during mid-2020 and the associated consequences can be a reference for policymaking in many places to manage various mass gatherings such as religious events in the face of other new epidemics.

## Method and materials

We utilize a quantitative approach, namely, Synthetic Control Method (SCM) for a comparative study to assess the impact of mass gathering on the local spread of COVID-19 in the local communities of Hong Kong. This analysis has a premise that the only way we can assess the influence of mass gatherings on the local transmission of COVID-19 is to compare the policy outcome with an appropriate counterfactual control unit [29]. In reality, it would be difficult to find a city sufficiently similar to Hong Kong to act as the control unit. By selecting a combination of possible comparison units that is most representative of Hong Kong’s characteristics before the interventions, SCM suggests a systematic way of reproducing the counterfactual [30, 31]. To achieve this, an epidemiological database and an aggregator of city-level demographic, socioeconomic, and climatic data for more than 200 Chinese cities (including cities in the mainland and Macau) were cross walked. Based on this combined dataset, an SCM was then employed to simulate a "synthetic Hong Kong" as the counterfactual that is not affected by mass gatherings and public demonstrations. Controlling for other confounding factors that could affect the COVID-19 transmission, such as city-level socioeconomic characteristics and climatic conditions, the synthetic control obtained from this simulation shares similar pre-intervention characteristics with Hong Kong. By comparing the actual number of confirmed cases with the estimated outcomes in our counterfactual model, we can empirically assess the impact of mass gatherings on the transmission of COVID-19 in Hong Kong.

Mass gatherings, especially public gatherings and demonstrations re-emerged after various social distancing policies, including the prohibition on group gathering regulation (POGGR), were lifted in June. These mass gatherings have a higher level of social mixing and thereby pose a higher risk of virus spreading, compared to routine activities (e.g., school, work, shopping, entertainment, etc.) and social gatherings, Our *hypothesis* is that mass gatherings happened after the relaxation of social policy contributed to the third wave of COVID-19 outbreaks in Hong Kong since early July.

Small-size gatherings (less than 50 people) after the relaxation of POGGR on June 19 may also confound the influence of mass gatherings on the transmission of COVID-19. In our empirical model, we apply two approaches to rule out this confounding effect. First, the potential donor units (i.e., all mainland cities) in our SCM model also adopted restrictions on public gatherings during the entire study period. According to the guidelines announced by the Chinese authorities, public gatherings were to be avoided in principle for medium and high-risk areas and only be held prudently in regions with low-level risk [32]. All urban and rural communities were to strictly manage community activities and restrict large-scale public gatherings until the end of the pandemic [33]. In fact, as of July 2020, mass gatherings of more than 200 people were still prohibited in most mainland cities. Therefore, the synthetic control constructed from these donor units can rule out the confounding effects of the relaxation of POGGR (up to 50 people as of June 19) because it theoretically allows for mass gatherings of more than 50 people (normally up to 200 people), yet Hong Kong only allows for gatherings of 50 people. If Hong Kong still exhibits a higher (and increasing) infection rate than the synthetic control during the intervention period, it is unlikely to have been caused by these small-size public gatherings. Second, we conducted robustness tests by changing the intervention point in the simulation model. Specifically, we use each of the 10 days following June 20 as the intervention point (i.e., June 20, 21, 22…), which gives us a longer control period to partially account for the impact of the relaxation of POGGR on June 19. Allowing for a longer control period that also takes into account the incubation period and the testing lags in COVID-19 detection.

Another advantage of using a longer control period is that the model can evaluate the effectiveness of those social distancing policies implemented until mid-June. In fact, the daily reported number of new cases of COVID-19 in Hong Kong had stabilized and remained low (almost 0 local cases in a single day, see Fig 1) until the beginning of July. This suggests the various social distancing policies implemented before the occurrence of the large-scale public gatherings and demonstrations in June and July were effective in containing local transmission. The empirical model elaborated below can more accurately identify any nodes of exceptional increases in daily new confirmed cases during the control period.

Nonetheless, we recognize that this study has significant limitations. There are disparities in the social and political environment of Hong Kong and mainland cities, for example, Hong Kong has a unique political system and economic autonomy, and different levels of citizen compliance to government policies as well as public trust in government. Such embedded discrepancies might affect our estimation of the impact of mass gatherings’ impact on COVID-19 transmission. While acknowledging the importance of capturing these factors in reproducing the synthetic control, we are not able to control them accurately in the SCM model due to their time-invariant nature. Instead, we introduce an extended pre-intervention period in our model and simulate the synthetic control with a border span of predictors over that period. Therefore, the “synthetic HK”, which is constructed by the optimal weighted combination of potential donor cities, is simulated with the greatest possible matching for all predictors and variables of interest, and any unobservable factors can be implicitly captured variables.

### Data and data sources

This research aggregates a COVID-19 database with city-level socioeconomic, demographic, and climatic data for more than 200 Chinese cities to construct a counterfactual context for comparative analysis to examine the impact of Hong Kong’s mass gatherings, public demonstrations in particular, on the local outbreak of COVID-19. The existing literature suggests that the development of pandemics is catalyzed by multiple factors. Therefore, we incorporate a number of predictors into our SCM models, including epidemiological variables (i.e., the number of new/total confirmed cases), city-level basic characteristics, and climatic parameters. Additionally, three epidemiological variables are also included as predictors to estimate the trends of infection in the14-day before intervention and preclude any confounding factors associated with the systematic discrepancies between the simulated synthetic control and HK. They are, the average of total case numbers, average of total cases per 10,000 persons (taking population size into consideration), and sum of daily new cases, which are calculated for the 14-day period before the intervention point. Our SCM models take the indicators of new and total cases daily to capture the outbreak situation scenario and use other city-level variables as predictors. All the city-level data is gathered from several sources.

#### City-level COVID-19 data

The primary data of our SCM analysis is the city-level daily epidemiological data of the COVID-19 outbreak. A China Data Lab collected the daily epidemiological data between June and July and the data are openly accessible on Harvard Dataverse. We retrieved data from this dataset on the daily number of new and total cases for each prefecture-level city in the mainland, as well as for Hong Kong and Macau.

#### Demographic and socioeconomic data

A number of studies have suggested that regional socioeconomic factors, such as the concentration level of population, total factor productivity, the degree of unemployment, accessibility to healthcare influence how fast and how far infectious diseases are transmitted [10, 34–41]. It is, therefore, necessary to consider demographic characteristics (average population, number of households, population density). Demographic statistics of each city were derived directly from the latest version of the China City Statistical Yearbook (2019), which provides information in 2018. To calculate the population density of these cities, we utilized geographic data on the total land area from the same data source. For data on Hong Kong and Macau, we used 2018 demographic data from the official Statistical Yearbooks provided by their Socioeconomic and Statistics Departments. We incorporated several predictor variables to control for socioeconomic factors in our empirical model, including the level of economic development (represented by total GDP, per-capita GDP) and public health capacity (represented by the number of hospitals, and the numbers of doctors and hospital cots per 10,000 people).

#### Natural meteorological parameters

Previous research has demonstrated that weather conditions and air quality potentially play a role in the spread of COVID-19 [41, 42–47] and other infectious diseases [38, 48–50]. Hence, this research considers various daily natural meteorological factors, including air quality index (AQI), average temperature, wind speed, and relative humidity, to simulate the counterfactual scenario and estimate case numbers. These factors are all proven to impact the transmission of an epidemic in the previous literature, but many studies on COVID-19 transmission do not control for their effects. Simulated by the SCM models, the constructed synthetic control in the absence of mass gatherings has similar climate conditions to Hong Kong; therefore, we are able to control for any influence on the estimated case number of COVID-19.

Climatic parameters for cities in mainland China were derived from the China Meteorological Data Service Centre, which provides hourly records of common meteorological factors such as temperature, relative humidity as well as wind speed at each meteorological observatory station. We calculated daily numbers by averaging the hourly data for each variable. For each city with epidemiological data, we used an Empirical Bayesian Kriging interpolation to compute the values of the three meteorological elements in ArcGIS. Kringling is a statistical approach for predicting the best possible location in a geographic region, which has been widely used in meteorological applications, agriculture, geoscience, and many other disciplines because of its minimized prediction error. Because it offsets the deviations resulting from the semivariogram model, the Empirical Bayesian Kriging method is more resilient than conventional Kriging techniques [51]. Therefore, we adopt the Empirical Bayesian Kriging as the interpolation method of meteorological parameters in this study; the parameters in the model are the default settings of the software. The daily climatic parameters of Hong Kong are collected from the Hong Kong Observatory, and those of Macau SAR are extracted from the Meteorological and Geophysical Bureau.

We captured the dynamics of air quality in each city by Air Quality Index (AQI), which is measured based on the number of six atmospheric pollutants (e.g., SO_2_, PM2.5, PM10) detected at all inspection stations within the city border. The source of Chinese cities’ air quality data is from Harvard Dataverse. Each record includes information on the daily average, maximum, minimum, and standard deviation value of AQI. In this study, we use the daily average AQI to represent the air quality conditions of each city. Meanwhile, World Air Quality Index (WAQI) project provides daily AQI data of all air quality monitoring stations in Hong Kong and Macau. The values of daily AQI in Hong Kong (or Macau) were calculated by averaging the daily data of all air quality monitoring stations in the region.

### Model specification

Through optimal weighted linear simulations using data from over 200 Chinese cities, we simulate a "synthetic Hong Kong" that has many of the same features as the actual Hong Kong. We estimate COVID-19 transmission patterns using such synthetic control for a counterfactual scenario in which there were no public gatherings and demonstrations in Hong Kong. Based on aggregated epidemiological data from chosen cities, we estimate the trend in the daily numbers of new and total cases in this counterfactual circumstance (i.e., donor units). We may analyze the influence of mass gatherings on COVID-19 transmission in local communities in Hong Kong by comparing the real epidemiological trajectory recorded in the city with simulated trends, and therefore measure the effectiveness of the HK government’s social distancing regulations.

As discussed above, our SCM model uses a long control period starting on May 1 and ending on June 19 to eliminate the confounding effects of all other events that happened during the same period as the public gatherings and demonstrations studied. As the first large-scale political gathering in our study period happened in Hong Kong on June 20, the beginning of the intervention period is designated as June 20. During the intervention period, a series of public gatherings and demonstrations occurred (see Fig 2). We expect that significant differences in infection numbers between the synthetic control and Hong Kong may not be observed for days immediately following June 20. This is because of the existence of a median incubation period of 5 days and a potential testing and reporting lag of 1–2 days. People infected during public gatherings and demonstrations may not be reported as confirmed cases until several days after the protest. Taking into account these potential lags, we also conduct robustness checks by using each of the 10 days following June 20 as different intervention points (i.e., June 21, 22, 23…).

The SCM method has been widely used to assess the impact of political reforms and various economic policies. [29, 52–57]. Rather than selecting a real city as the counterfactual for comparison, our SCM combines several comparison units (i.e., over 200 Chinese cities) from the donor city pool by an optimum weight of each donor cities, to construct one a synthetic Hong Kong as counterfactual. The inherent advantage of the SCM is that it offers a data-driven method for determining a matching counterfactual used for comparative analysis. As a result, issues of ambiguity, endogeneity, and self-selection biases can be eliminated.

The following is an illustration of our SCM model: *Y*_*jt*_ is defined as COVID-19 infections diagnosed in city *j* at time *t*. There are *J*+1 cities, with Hong Kong as the first city (*j* = 1) being subject to intervention (i.e., the occurrence of mass gatherings in the form of public gatherings and demonstrations) after time *T*_0_ (i.e., June 20), and the remaining *J* cities (selected cities in China) are donor cities for simulating the “synthetic Hong Kong”. The number of cases for city *j* with the occurrence of mass gatherings is set as $Y_{jt}^{I}$ and the estimated infection trend in the synthetic control is set as $Y_{jt}^{N}$, the later hypothetically assume that mass gatherings had not occurred at time *t*∈[*T*_0_+1,*T*]. Thus, $Y_{jt}^{I}$ is observable as

$$Y_{jt}^{I}=Y_{jt}^{N}+\tau_{jt}D_{jt}$$

where *τ*_*jt*_ is the impact of mass gatherings on COVID-19 cases number for city *j* at time *t* (i.e., from June 20 onwards). As Hong Kong, that is, *j* = 1, is the only city that affected by mass gatherings, *D*_*jt*_ is the indicator written as the following:

$$D_{jt}=\begin{array}{cc} 1 & for\;j=1,t>T_{0}\\ 0 & otherwise \end{array}$$

*D*_*jt*_ indicates whether city *j* was influenced by public gatherings in period *t* (i.e., from June 20 onwards). *D* takes value 1 for the treated unit (j = 1), and 0 otherwise. For *t*>*T*0, ${\tau_{1t}=Y}_{1t}^{I}-Y_{1t}^{N}$ gives the net effect of mass gatherings on the increase of COVID-19 infections.

The objective is to assess the value of $\tau_{1T_{0}+1},\;\tau_{1T_{0}+2},\ldots ,\tau_{1T}$.

In accordance with the algorithm stated in [53], we assume that $Y_{jt}^{N}$ is given by a factor model

$$Y_{jt}^{N}=\delta_{t}{+\theta_{t}Z}_{j}+\lambda_{t}\mu_{j}+\varepsilon_{jt}$$

where *Z*_*j*_ = [*Z*_*j*1_,…,*Z*_*jr*_]′ is a (*r*×1) vector of predictors that are observable and irrelevant to the occurrence of mass gatherings, as presented in Table 2. *θ*_*t*_ = [*θ*_1*t*_,…,*θ*_*rt*_] is a (1×*r*) vector of parameters. Also, a (1×*k*) vector of common factors which are unobservable is captured by *λ*_*t*_ = [*λ*_1*t*_,…,*λ*_*kt*_]. A *k*×1 vector of unknown factor loadings is represented by *μ*_*j*_ = [*μ*_*j*1_,…,*μ*_*jk*_]′. Unobservable ephemeral impacts with zero mean are depicted by the error terms *ε*_*jt*_. A constant factor loading is described by *δ*_*t*_., a common factor which is unknown.

The actual case number reported in Hong Kong are observable and we have $Y_{jt}^{I}=Y_{1t}$, but we have no way to observe how many infected cases would be under the conditions of the synthetic control $Y_{1t}^{N}$ within the period from *T*_0_+1 tp *T*. In order to approximate the value of $Y_{1t}^{N}$, we utilized the procedure stated in [52]. A (*J*×1) vector of weights *W* = [*w*_2_,…,*w*_*J*+1_]′ is simulated so that we can have $\sum_{j=2}^{J+1}w_{j}=1$, and *w*_*j*_≥0 for *j* = 2,…,*J*+1.

By using a weighted average of donor cities with observed outcomes and covariates among the time period before the intervention point, we estimate *Y*_1*t*_ and *Z*_1_ for the phase before the occurrence of intervention, i.e., *t*≤*T*_0_. Thus, *W* = (*w*_2_,…,*w*_*j*+1_) is computed for *t*∈[1, *T*_0_], such that

$$Y_{1t}=\sum_{j=2}^{J+1}w_{j}\;Y_{jt}$$

$$Z_{1}=\sum_{j=2}^{J+1}w_{j}\;Z_{j},$$

Once *W* is estimated, the effect of intervention (i.e., mass gatherings) at *t* = *T*_0_+1, *T*_0_+2,… can be captured by

$$\tau_{1t}=Y_{1t}-\sum_{j=2}^{J+1}w_{j}\;Y_{jt}$$

*W* is the optimum weight for the combination of control units (i.e., donor cities), which minimizes the disparities of predictor and outcome variables between the reality and the counterfactual among the pre-intervention phase. We utilized the technique described in [53] to get the optimal weights, which technically minimizes the mean squared prediction error of all predictors and outcome variables over the pre-intervention period.

## Results

After data cleaning, 282 Chinese cities were included in the donor pool for constructing the synthetic control. In our baseline model in Panel A, we designate June 20, one day after the lift of POGGR, as the beginning of the intervention period, when Hong Kong began observing mass gatherings including a series of public gatherings and demonstrations. Table 1 demonstrates the distribution of sample weights of the selected comparison cities for the synthetic control simulated in Panel A. We constructed the synthetic control and estimated the trends of the daily numbers of COVID-19 new and total cases in Model 1 and Model 2. The synthetic control obtained in Model 1 was constructed using 6 donor cities, while that obtained in Model 2 was from 4 donor cities. Table 2 demonstrates the balance of predictor variables during the pre-intervention period, indicating that the characteristics of the synthetic control are very similar to those in Hong Kong before the occurrence of public gatherings and demonstrations. This suggests that our synthetic control simulated from the SCM models is a good fit with the real Hong Kong in the pre-intervention period.

**Table 1:** Distribution of sample weights in the donor pool for synthetic Hong Kong.

Panel A: intervention point on June 20			
Model 1 (Fig 3)		Model 2 (Fig 4)	
Variable of interest: new cases		Variable of interest: total cases	
Name of the donor city	Weight in the synthetic control	Name of the donor city	Weight in the synthetic control
Jilin	0.265	Jilin	0.47
Suihua	0.261	Shanghai	0.427
Shanghai	0.221	Beijing	0.192
Macau	0.159	Wuhan	0.089
Beijing	0.082		
Huanggang	0.012		

**Table 2:** Pre-intervention balance of predictor variables in Panel A (June 20 as the intervention point).

Pre-intervention characteristics during the control period (May 1 –June 19)			
		Model 1	Model 2
	Real HK	Synthetic HK	Synthetic HK
# of new cases in the past 14 days	22.14	6.90	12.5
Preintervention 14-day (June 6- June 19) average of daily total case numbers	1,111	271.81	1,078.14
Preintervention 14-day (June 6- June 19) average of daily number of total cases per 10,000 people	1.49	1.47	1.18
Preintervention 14-day (Jun 6- Jun 19) sum of new confirmed cases	25	19.43	24.83
Region GDP (million yuan)	2,398,046	1,069,235	1,750,217
# of persons per sq.km (population density)	6,736.89	4,238.87	1295.06
# of Hospital Cots per 10,000 persons	54.27	59.83	90.98
# of Doctors per 10,000 persons	19.66	39.12	52.75
Average Temperature (°C)	28.31	20.18	20.03
Relative Humidity (%)	81.92	71.30	72.29
Wind Speed (m/s)	5.29	2.76	2.68
Air Quality Index (AQI)	44.28	45.21	52.03

**Fig 3:**
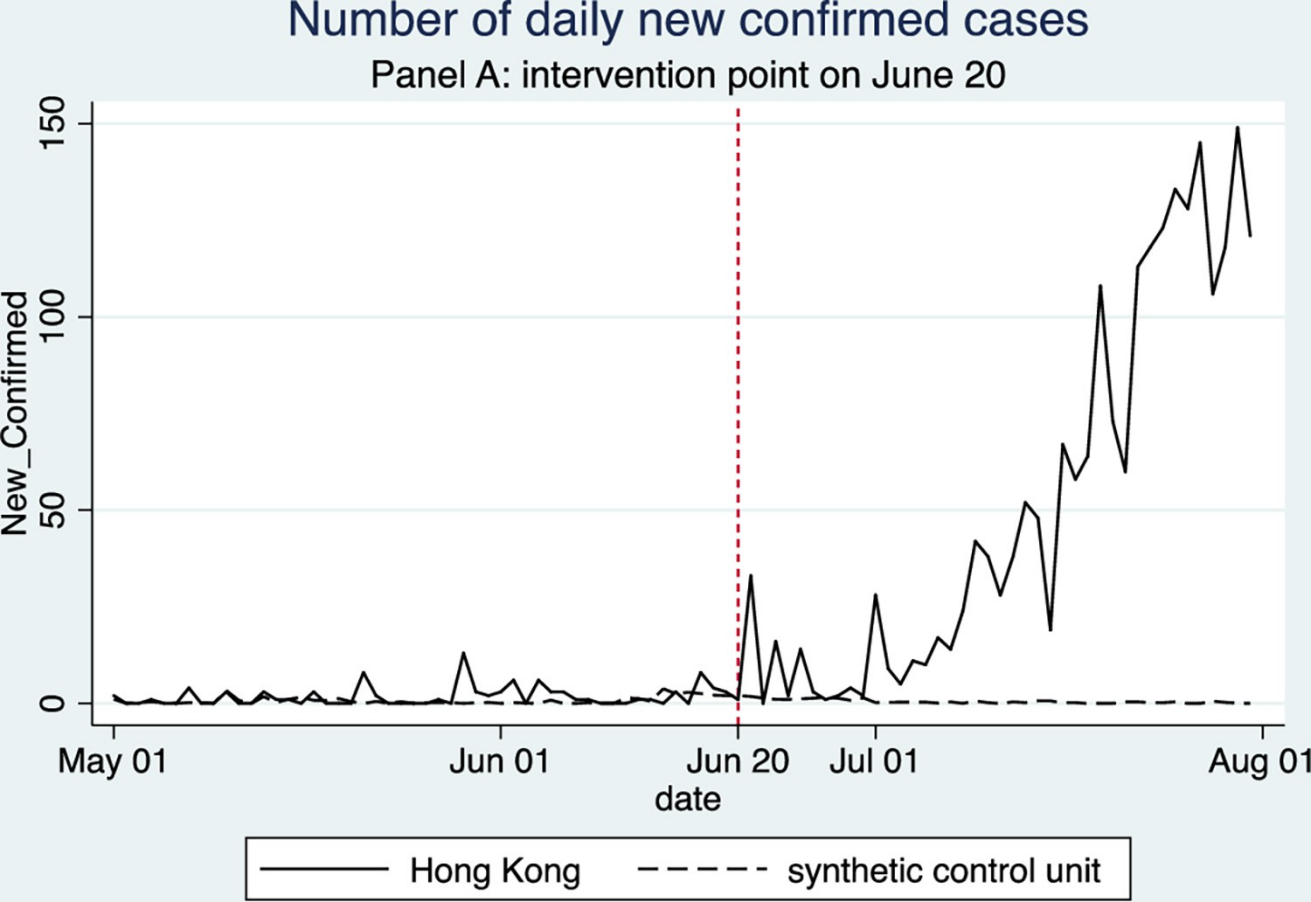
Impact of mass gatherings (after the lift of POGGR) on COVID-19 new cases.

Fig 3 shows the comparison between the actual and simulated trends of COVID-19 new cases confirmed on a daily basis. The residual between the two trajectories after the date of intervention can be interpreted as the impact of mass gatherings after the relaxation of social distancing order, i.e., POGGR, on COVID-19 transmission in local communities. As shown in the figure, Hong Kong saw a much higher number of daily new infections than the synthetic control that models a hypothetical situation assuming no mass gathering happened. This indicates that public gatherings and demonstrations in Hong Kong contributed to the increase in new cases on a daily basis. In the absence of public gatherings and demonstrations, Hong Kong would have no more than 2 new cases registered on any single day from June 20 until the end of July. To explain this in another way, we can calculate the sum of new infections by adding up these predicted daily new cases. For the first 14 days after the intervention date, Hong Kong would have observed around 16 new cases if the POGGR had not been lifted, while the actual number of new cases was 120 during this period. After the first two weeks, the outbreak was under control in the synthetic control, with at most 1 new case confirmed in any single day throughout the rest of July. However, the actual infection numbers in Hong Kong continued to rise dramatically, and as many as 2145 new cases were recorded from June 20 to July 31, significantly more than the 23 new cases estimated in the synthetic control during the same period. This confirms that the protest and demonstration activities in late June and early July contributed to a considerable growing trend of daily new cases.

**Fig 4:**
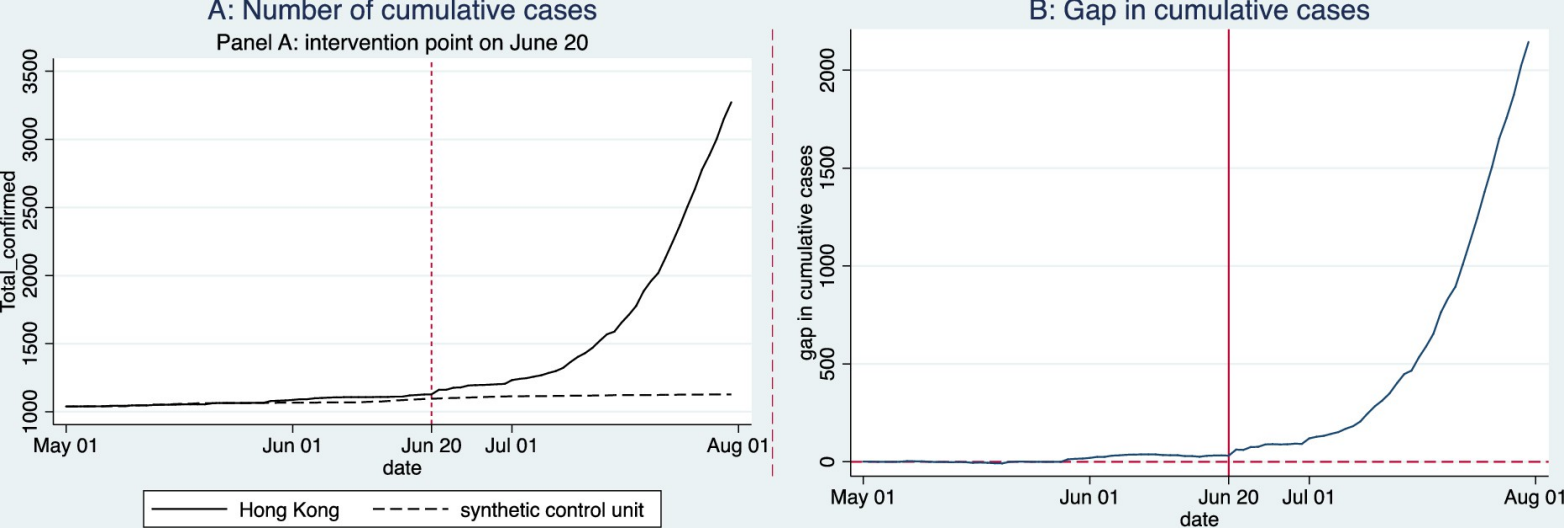
Impact of mass gatherings (after the lift of POGGR) on COVID-19 total cases.

Fig 4A shows the trends of total infections reported and estimated in our synthetic control. The visual comparison suggests that a significant increase in the number of cumulative infections may be attributed to public gatherings and demonstrations during the period from June 20 to the end of July. Before the intervention point (June 20), the figure of total infections in both scenarios (i.e., with and without mass gatherings) is similar, but after June 20, the gap between the two lines widens sharply. This gap is also represented as a line graph in Fig 4B, indicating that public gatherings generally increase COVID-19 infections in the local communities of Hong Kong. The results suggest that if Hong Kong had not been affected by public gatherings and demonstrations after June 20, the outbreak would have stayed at a moderate level, with just around 1128 total cases by July 31, while actual infections exceeded 3000 cases. More importantly, the actual growth rate of infections in the city is significantly higher than that of the counterfactual model. During the period from June 20 to July 31, the total number of infections increased by 2144 cases in Hong Kong, and the average daily growth rate of total cases was 2.63%.

Calculating the average daily growth rate of total infections: Hong Kong has 1128 total cases on June 20 compared to an estimated number of 1125 total cases in the synthetic control. On July 31, Hong Kong counts 3272 cases while the synthetic control has 1164 cases, in total. From June 20 to July 31, The average daily growth rate in Hong Kong is denoted by r and can be computed from 1128 (1+ r)^41^ = 3272. Similarly, the average daily growth rate in the synthetic unit rc can be computed from 1125(1+ rc)^41^ = 1164.

In our counterfactual model, however, there was an increase of only 32 cases in the number of total infections; the average daily growth rate was a mere 0.07%. Our results suggest that, if Hong Kong had not been affected by mass gatherings after June 20, the city could have controlled the increase of new infections and kept the number of total infections at a low level.

**Fig 5:**
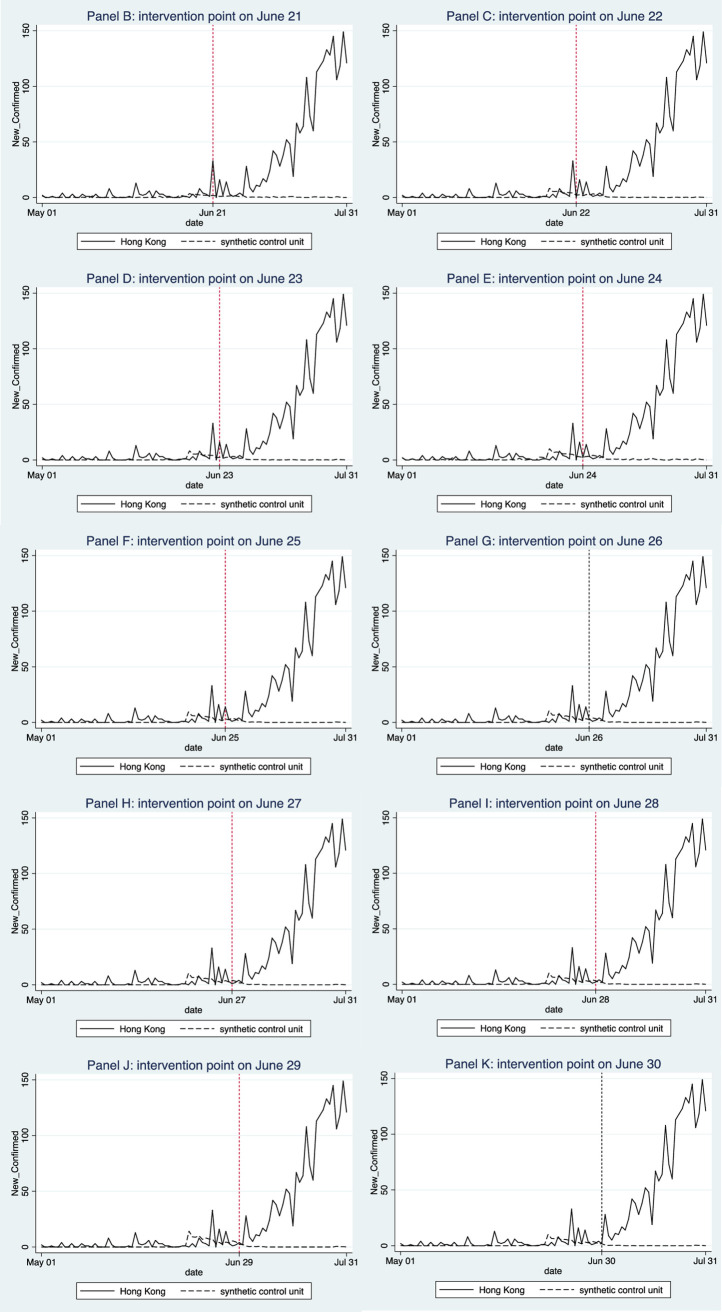
Impact of mass gatherings (after the lift of POGGR) on the number of new cases of COVID-19 (with intervention point changed). When we changed the intervention point (i.e., the starting date of lifting of public gathering restrictions in Hong Kong), we obtained different estimates of new cases in the respective synthetic controls. However, the adjusted results still show that the actual number of daily new cases of COVID observed after the lift of public gatherings and the occurrence of and demonstrations is, in general, higher than the counterfactual estimated in the synthetic controls obtained in all the Panels. Except for Panels I, J, and K, other Panels using an earlier intervention point indicate that the counterfactual estimates of new case numbers are initially close to the actual number. After some fluctuations, the synthetic trends stabilize in the later stage, while the actual infections numbers grew sharply. If we impose longer lags (as in Panel I, J, and K), meaning that we have a later intervention point, the gap between infection numbers under the two scenarios becomes more significant even in the early stage of the intervention.

To cover a longer control period and to consider the long incubation period of COVID-19 (i.e., delayed symptom onset after infection), as well as delays in testing and reporting, we changed the intervention point to conduct robustness tests. Here we focus on Model 1, which simulates the trend in daily new cases. If the results in Model 1 are robust, we can infer that the results of Model 2 are also robust. Using the same predictors and outcome variable, we use each of the 10 days after June 20 as the intervention point (i.e., June 21, 22, …, 30) in each panel to re-examine our estimation model. Fig 5 shows the results obtained in each Panel with different intervention points. The sample weights of comparison cities that construct the synthetic Hong Kong are provided in the S1 Appendix.

Table 3 summarizes the magnitude of the impacts during the 10-day and 30-day periods after the intervention point. In our baseline configuration of the SCM model (Panel A), which uses the start of protest activities (June 20) as the intervention point, the size of the impact of mass gatherings in the first 10-day period after the POGGR relaxation is a growth of 62 new cases. The 30-day impact is an increase of 737 new cases. These increases in new cases due to the impact of mass gatherings account for 87.58% and 97.23% of the total new cases accumulated over the respective time periods. We also conduct robustness checks for these results by extending the control period to capture the lags in the onset of symptoms and testing delays, where we use each of the 10 days after June 20 as the intervention point. When considering shorter lags (1–5 days), we estimate that, on average, protest activities contribute to an increase of around 57 new infections (or 75% of total new cases) over the 10-day post-intervention period and an increase of around 961 new infections (or 97.66%) over the 30-day period. When we consider longer lags (6–10 days), the impact is more profound, with an increase of 101 new cases (or 91.82%) and 1553 new cases (or 99.36%) over the 10-day and 30-day periods, respectively. Therefore, we conclude that public gatherings and demonstrations in Hong Kong in late June and early July contributed significantly to the new wave of COVID-19 infections that began in early July.

**Table 3:** Summary of the impact of mass gatherings (after the relaxation of social distancing policy) on the increase of new cases during the 10-day and 30-day periods after intervention, taking June 20 and each of the 10 days afterward as the intervention point.

		Baseline	Robustness tests									
		Panel A	Panel B	Panel C	Panel D	Panel E	Panel F	Panel G	Panel H	Panel I	Panel J	Panel K
Intervention point		June 20	June 21	June 22	June 23	June 24	June 25	June 26	June 27	June 28	June 29	June 30
10-day period after intervention	Hong Kong	76	77	72	81	70	79	75	89	102	124	162
	Synthetic control	14	13	21	18	24	17	15	13	9	8	4
	Difference	62	64	51	63	46	62	60	76	93	116	158
30-day period after intervention	Hong Kong	758	830	857	970	1072	1193	1312	1437	1581	1685	1799
	Synthetic control	21	20	25	22	34	17	16	13	9	8	5
	Difference	737	810	832	948	1038	1176	1296	1424	1572	1677	1794

## Discussion

This study investigates whether the relaxation of social distancing policies and the subsequent occurrence of public gatherings contributed to a new wave of local outbreak (also known as the third wave) in Hong Kong. After more than two years into the pandemic, the global scientific community has gained significant understanding of COVID-19 regarding the relationship between small group gatherings and viral transmission, especially in indoor settings. However, previous studies leave a research gap in terms of how mass gatherings in public places, which generally have lower perceived risks but higher uncertainties, could influence local transmission. By examining the series of public demonstrations that happened in Hong Kong following its relaxation of the Prohibition on Group Gathering Regulation (i.e., the POGGR), this study provides important empirical evidence for the implementation of social distancing policies in limiting mass gatherings and controlling virus transmission.

Various public gatherings and demonstrations took place in Hong Kong from June 20, 2020 onwards. These events were collectively influenced by the enactment of the NSL in the city and the coincidental relaxation of POGGR. Although people usually were wearing masks during public gatherings and demonstrations, these activities still tend to be highly crowded with increased physical contact, creating openings for virus transmission and increasing the rate of COVID-19 infection. Some have suspected that the third wave was a result of the loosened quarantine requirements, especially the quarantine exemptions for special overseas arrivals such as seafarers [58]. Government anti-epidemic advisers and medical experts estimate that the new wave of outbreaks originated from a number of people who were exempted from quarantine and introduced into the community via taxi drivers. However, the majority of cases during the investigation period (over 83%) were reported as local infections [59], implying that the lifting of social distancing order played a role in influencing the transmission during Hong Kong’s third wave. Using an aggregated dataset of epidemiological, city-level meteorological and socioeconomic data, our SCM modeling results clearly evidence that public and social mass gatherings that occurred after the relaxation of social distancing policy had a sizable impact on COVID-19 transmission in Hong Kong’s local communities. Our findings indicate that from June 20 to July 31, 2020, Hong Kong observed an infection rate, as captured by the mean daily growth rate of total cases, substantially greater than that estimated by the counterfactual control unit (2.63% versus 0.07%). This transmission rate has led to exponential growth in new cases. We also estimate that public gatherings and demonstrations increase the number of new infections by 62 cases (or 87.58% of total new cases) over the 10–day period after they first occurred on June 20 and by 737 cases (or 97.23%) over the 30-day period. These findings are in line with local clinical experts who argued that the propagated outbreak in the third wave derived from increased social gatherings during dragon boat festival (June 25) and July 1 holiday [60].

Based on our aforementioned findings, this research provides important guidance for *local* anti-pandemic policymaking in Hong Kong. We recommend that it is vital for the Hong Kong government to strengthen its law enforcement to caution any large-scale public gatherings during the pandemic, including public gatherings and demonstrations. Our findings suggest that participants in large-scale gatherings are susceptible to infection when the virus has not been completely controlled. Based on Hong Kong’s experience during the third wave, the relaxation of social distancing restrictions in late June 2020 created a loophole for large-scale public gatherings. In particular, the lifting of the POGGR on June 19 created opportunities for small- or medium-sized gatherings and even large-sized demonstrations (though unpermitted) to occur. Our empirical findings corroborate that these gatherings have led to a resurgence in local COVID-19 infections. Also, social distancing policies are of great importance in limiting mass gatherings and managing the risk of local transmission of COVID-19 when herd immunity has not yet gained.

Hence, in this third wave of Hong Kong’s local outbreak, it was crucial to tighten restrictions on mass/group gathering promptly to curb the transmission of potential community infections and alleviate pressure on the public health system in Hong Kong. In fact, in August 2020, Hong Kong tightened the POGGR to a limit of 2 people, and later lifted this to 4 people in September 2020. As a result, the third wave gradually came under control. Acknowledging the uncertainty of the COVID-19 pandemic and the unique political and social challenges in Hong Kong, the government departments should commit to containing the pandemic for public society and welfare. The health department should attentively assess and monitor risks stemming from any mass gatherings and prompt adjustment of social distancing policies, accordingly. Public leaders of other functional constituencies should cooperate to address the root causes of social unrest in society, improve social welfare and rebuild trust in government among citizens. In such a way, the effectiveness of the government’s anti-pandemic policies can be guaranteed.

Our findings on the significant impact of mass gatherings on the transmission of COVID-19 in Hong Kong also provide strong evidence for the necessity of implementing social distancing policies *in other parts of the world*, especially in places where large-scale gathering events are also being experienced. The tragedy of the deadly 2022 Halloween Crush in South Korea has made the entire nation mourn, yet its immediate impact on the possible eruptive COVID-19 new cases may cause the nation to suffer much graver losses. In the face of other novel infectious diseases (NID) in the future, social distancing policies, and particularly those restrictions on group gatherings, should be prioritized once the local outbreaks are observed, and should be maintained until the outbreak is completely curbed. Constantly changing social distancing orders will create confusion and inconvenience among the public, as well as hinder the effectiveness of prior social distancing efforts.

In conclusion, this research not only makes significant contributions to the scientific literature on the impact of mass gathering on the transmission of COVID-19, but also has important implications for policymakers in designing more effective policies to control the pandemic. *First*, by paying attention to public gatherings that often take place in outdoor environments where ventilation is usually better, our research can help complement existing empirical studies that focus on how gatherings in indoor settings may influence virus transmission. The findings of this study will help advance our understanding of the transmission patterns and sources, and thereby provide scientific guidance for managing the risks of mass gathering events. *Second*, the study in Hong Kong not only helps the local government improve its policymaking capacity to combat the pandemic, but also contributes to relieving pandemic situations in other places around the world that are also experiencing mass gatherings (e.g., large-scale religious events). Hence, our findings can generate useful insights on pandemic control for countries considering or starting to relax their restrictions on group gatherings to adapt to the new normal during a pandemic.

Lastly, it is noted that this study does not address the more transmissive SARS-CoV-2 variants that were later observed in multiple countries. This is because the earliest SARS-CoV-2 variant, i.e., Alpha, first appeared in the U.K. in November 2020 (for more information about the evolution of the SARS-CoV-2 virus, see WHO’s official declaration at https://www.who.int/en/activities/tracking-SARS-CoV-2-variants/ and Yale Medicine’s guide to the coronavirus variants at https://www.yalemedicine.org/news/covid-19-variants-of-concern-omicron.) and our study period is restricted to between May and July 2020. Moreover, our study period also does not reflect on the ongoing situation wherein many cities/countries in the world have gained substantial immunity to COVID-19. Nevertheless, the past anti-pandemic experiences are still important lessons for the future. This study examines how public gatherings can influence the transmission of contagious viruses when herd immunity is yet to form. This can help to provide crucial evidence for evaluating the use of social distancing policies against early-stage outbreaks of other novel infectious diseases in the future. Such evidence is pivotal for both the scientific community and the general public.

## Supporting information

S1 Appendix (DOCX)
