# Supplementary material for: The impact of mass gatherings on the local transmission of COVID-19 and the implications for social distancing policies: Evidence from Hong Kong
Source: PLoS One. 2023 Feb 1;18(2):e0279539. doi: 10.1371/journal.pone.0279539 (PMC9891527; doi:10.1371/journal.pone.0279539)
Supplement: S1 Appendix — (DOCX) [file pone.0279539.s001.docx]

**Appendix**

Donor cities and weights for synthetic controls in the robustness tests

| **Panel B** | | **Panel C** | | **Panel D** | | **Panel E** | |
| --- | --- | --- | --- | --- | --- | --- | --- |
| **City** | **Weight** | **City** | **Weight** | **City** | **Weight** | **City** | **Weight** |
| Shanghai | 0.292 | Macau | 0.294 | Chengdu | 0.31 | Changdu | 0.25 |
| Macau | 0.265 | Beijing | 0.193 | Macau | 0.254 | Macau | 0.243 |
| Longnan | 0.124 | Longnan | 0.151 | Beijing | 0.206 | Beijing | 0.216 |
| Shuangyashan | 0.078 | Shuangyashan | 0.146 | Shanghai | 0.146 | Dazhou | 0.154 |
| Beijing | 0.075 | Dazhou | 0.1 | Changdu | 0.058 | Shuangyashan | 0.128 |
| Guigang | 0.059 | Guigang | 0.074 | Wuhan | 0.016 | Wuhan | 0.009 |
| Shantou | 0.051 | Shangrao | 0.023 | Suqian | 0.007 |  |  |
| Wuhan | 0.016 | Wuhan | 0.019 | Xiaogan | 0.005 |  |  |
| Changdu | 0.037 |  |  |  |  |  |  |
| Xiaogan | 0.003 |  |  |  |  | Wuhan | 0.015 |
| **Panel F** | | **Panel G** | | **Panel H** | | **Panel I** | |
| **City** | **Weight** | **City** | **Weight** | **City** | **Weight** | **City** | **Weight** |
| Macau | 0.262 | Chongqing | 0.319 | Changdu | 0.3 | Macau | 0.393 |
| Beijing | 0.216 | Macau | 0.29 | Macau | 0.297 | Shenzhen | 0.305 |
| Shanghai | 0.134 | Beijing | 0.24 | Beijing | 0.228 | Beijing | 0.229 |
| Shantou | 0.19 | Changdu | 0.151 | Chongqing | 0.134 | Shanghai | 0.041 |
| Shuangyashan | 0.073 |  |  | Xiaogan | 0.02 | Wuhan | 0.015 |
| Changdu | 0.056 |  |  | Suqian | 0.015 | Dongying | 0.012 |
| Chongqing | 0.046 |  |  | Wuhan | 0.007 | Nanjing | 0.004 |
| Wuhan | 0.016 |  |  |  |  |  |  |
| Chuzhou | 0.005 |  |  |  |  |  |  |
| Huanggang | 0.001 |  |  |  |  |  |  |
| **Panel J** | | **Panel K** | |  | |  | |
| **City** | **Weight** | **City** | **Weight** |  |  |  |  |
| Shanghai | 0.464 | Shenzhen | 0.373 |  |  |  |  |
| Macau | 0.282 | Macau | 0.337 |  |  |  |  |
| Changdu | 0.102 | Beijing | 0.23 |  |  |  |  |
| Tianjin | 0.055 | Shanghai | 0.042 |  |  |  |  |
| Haidong | 0.048 | Wuhan | 0.013 |  |  |  |  |
| Beijing | 0.022 | Nanjing | 0.005 |  |  |  |  |
| Wuhan | 0.014 |  |  |  |  |  |  |
| Shannan | 0.004 |  |  |  |  |  |  |
| Chongqing | 0.002 |  |  |  |  |  |  |
